# AI-assisted autonomous learning and reduced academic accomplishment in vocational higher education: the mediating role of hardiness

**DOI:** 10.3389/fpsyg.2026.1848291

**Published:** 2026-06-29

**Authors:** Wenli Wang, Qiuyan Zhang

**Affiliations:** 1 Human Resources Department, Xiamen Institute of Technology, Xiamen, China; 2 Faculty of Education and Livelihood, Xiamen City University, Xiamen, China

**Keywords:** academic burnout, AI-assisted autonomous learning, hardiness, psychological resources, reduced academic accomplishment

## Abstract

As generative AI tools become increasingly embedded in students’ everyday study routines, a key question in higher education is how AI-assisted autonomous learning relates to students’ academic adaptation. Existing research has largely emphasized the benefits of AI for efficiency, access to information, and personalized support, whereas less attention has been paid to its potential associations with students’ psychological resources and self-evaluative academic outcomes. Drawing on a psychological resource perspective, this study examined the relationships among AI-assisted autonomous learning, hardiness, and reduced academic accomplishment. Survey data were collected from 1,264 students at a vocational college in China and analyzed using structural equation modeling. The results showed that AI-assisted autonomous learning was negatively associated with hardiness and positively associated with reduced academic accomplishment. Hardiness was also negatively associated with reduced academic accomplishment and partially mediated the relationship between AI-assisted autonomous learning and reduced academic accomplishment. The indirect effect accounted for a substantial proportion of the total effect. These findings extend research on AI in higher education by suggesting that AI-assisted learning may have implications beyond efficiency and convenience, particularly for students’ psychological resources and academic self-evaluation. The study contributes to understanding academic adaptation in AI-mediated learning contexts and offers implications for the responsible integration of AI into higher education.

## 1 Introduction

The rapid development of generative artificial intelligence (GenAI) has begun to reshape learning in higher education. Since the public release of large language models such as ChatGPT, artificial intelligence has moved beyond a frontier technology and become part of students’ everyday academic practice (Alavi et al., 2024; Jarrahi et al., 2022). In particular, AI tools can now provide immediate explanations, feedback, and content support during self-directed study, making AI-assisted autonomous learning increasingly visible in university learning contexts. Existing research has shown that artificial intelligence can improve learning efficiency, support personalized learning, and enhance access to educational resources (Jagtap, 2025). In some domains, such as foreign language learning, ChatGPT-based tools have also been found to strengthen students’ self-efficacy and positive learning experiences (Xu et al., 2024). These findings help explain why AI-assisted autonomous learning has gained rapid acceptance among university students. At the same time, this development raises an important question for higher education: beyond improving efficiency and access to information, how does AI-assisted autonomous learning relate to students’ longer-term academic adaptation?

This issue is particularly important in vocational higher education. Vocational college students are expected to develop academic knowledge, practical skills, and professional competence. Their learning often depends on repeated practice, problem solving, and active engagement with real or simulated tasks. In this context, students’ sense of academic accomplishment is closely related to their experience of effort, mastery, and competence development. AI tools may support these processes by providing timely assistance. Yet they may also reduce students’ opportunities for independent thinking and effortful problem solving when used as a substitute for learning.

Although the educational benefits of artificial intelligence have received growing attention, concerns about its potential risks have also increased. Scholars have pointed out that excessive reliance on artificial intelligence may weaken independent thinking, increase technological dependence, and reduce opportunities for autonomous problem solving (Hendrycks et al., 2023; Zhai et al., 2024). In learning contexts, students may become increasingly accustomed to instant answers and optimized solutions, which may reduce deep engagement with academic tasks and shift learning toward faster but more superficial processing. From this perspective, AI-assisted autonomous learning should not be evaluated only in terms of convenience or short-term performance gains. Rather, it may also influence how students cope with challenge, effort, and uncertainty in academic work. However, compared with the expanding literature on technology acceptance, academic performance, and short-term learning outcomes, much less attention has been paid to whether AI-assisted autonomous learning is associated with students’ psychological resources and their self-evaluation of academic functioning. This gap is important because academic adaptation in higher education depends not only on the availability of learning support, but also on the internal resources that help students persist, regulate effort, and interpret difficulty constructively.

The present study focuses on reduced academic accomplishment, a core dimension of academic burnout. Reduced academic accomplishment refers to students’ negative evaluation of their academic competence and learning effectiveness (Lian et al., 2005; Maslach and Jackson, 1981). It is especially relevant to AI-assisted learning because it reflects whether students experience learning outcomes as meaningful and self-generated. When students obtain answers through AI but do not feel that they have truly mastered the content, their sense of academic accomplishment may decline. This possibility is important for vocational college students, whose academic confidence is closely connected with skill development and professional identity.

Hardiness may provide a psychological mechanism for understanding this relationship. Hardiness refers to a relatively stable disposition characterized by commitment, control, and challenge (Kobasa, 1979). It helps individuals remain engaged, perceive difficulty as manageable, and respond adaptively to stress. In educational settings, hardiness has been shown to protect students from academic stress and burnout (Abdullahi et al., 2014; Amelia et al., 2025; Fauziah and Affandi, 2025; Nuradilla, 2024). However, hardiness may be less activated when learning environments reduce students’ opportunities to face challenges, regulate effort, and solve problems independently.

Against this background, the present study examines the relationships among AI-assisted autonomous learning, hardiness, and reduced academic accomplishment among vocational college students in China. The study aims to extend current research on AI in higher education beyond technology acceptance and learning efficiency. It focuses on students’ psychological resources and academic self-perception in AI-supported learning contexts. Specifically, this study investigates whether AI-assisted autonomous learning is associated with reduced academic accomplishment and whether hardiness mediates this relationship.

## 2 Literature and theoretical framework

### 2.1 AI-assisted autonomous learning in vocational higher education

AI-assisted autonomous learning refers to students’ active use of generative artificial intelligence tools to obtain information, complete learning tasks, solve problems, and regulate their learning process. Compared with traditional digital learning tools, generative AI has stronger interactive and generative functions. It can provide dialog-based responses, structured explanations, and immediate feedback (Cabero-Almenara and Barroso-Osuna, 2025; Xu et al., 2024). These features make AI tools useful in self-directed learning contexts. They allow students to access academic support beyond the classroom and to adjust learning strategies according to their immediate needs.

Previous studies have emphasized the educational potential of AI-supported learning. Generative AI can provide personalized guidance and timely feedback, which may improve learning engagement and academic support (Kasneci et al., 2023). A systematic review by Celik et al. (2022) also suggested that AI tools can enhance learning processes and reduce teachers’ workload. In vocational higher education, these functions may be especially valuable because students often face both academic and practice-oriented learning tasks. AI tools may help students understand concepts, organize materials, and prepare assignments. However, their value depends on whether students use them as scaffolds or as substitutes for active learning.

The potential risk is that students may become dependent on AI-generated answers. When AI tools complete too much of the learning process, students may reduce their own cognitive effort, reflection, and problem solving. This may be especially problematic in vocational learning, where competence is built through repeated practice and active engagement. Therefore, AI-assisted autonomous learning should be examined not only as a learning strategy, but also as a learning condition that may reshape students’ psychological adaptation.

### 2.2 Reduced academic accomplishment as an academic adaptation outcome

Academic burnout originated from the theory of occupational burnout and has been extended to educational contexts (Maslach and Jackson, 1981; Schaufeli et al., 2002). It generally includes emotional exhaustion, academic cynicism or misconduct, and reduced academic accomplishment. Among these dimensions, reduced academic accomplishment refers to students’ negative evaluation of their academic competence and learning effectiveness. It reflects the feeling that one is not learning well or not making meaningful progress. Therefore, it is closely related to students’ academic self-perception.

This study focuses on reduced academic accomplishment rather than academic burnout as a global construct. This focus is appropriate because reduced academic accomplishment is most closely linked to perceived competence. In AI-assisted learning contexts, students may complete tasks more quickly with AI support. However, efficient completion does not always produce a genuine sense of mastery. If students rely mainly on AI-generated content, they may experience a gap between task completion and personal competence. This gap may increase negative academic self-evaluation.

Previous research has shown that academic burnout is associated with poorer academic performance, stronger dropout intention, and lower mental health (Madigan and Curran, 2020). In digital learning environments, changes in learning structure may further influence burnout-related experiences. Dong et al. (2025) found that generative AI misuse was associated with lower learning motivation and higher emotional exhaustion. Although this evidence focuses more on emotional exhaustion, it suggests that AI use may influence burnout through psychological mechanisms. Reduced academic accomplishment therefore provides a precise outcome for examining the possible psychological cost of AI-assisted autonomous learning.

### 2.3 Self-determination theory as the main theoretical framework

Self-Determination Theory provides the main theoretical framework for this study. The theory proposes that autonomy, competence, and relatedness are basic psychological needs that support motivation, learning, and well-being (Ryan and Deci, 2000). Among these needs, autonomy and competence are most relevant to AI-assisted autonomous learning. Autonomy refers to students’ experience of volition and ownership in learning. Competence refers to students’ experience of effectiveness and mastery. When these needs are supported, students are more likely to show persistence, engagement, and positive academic self-evaluation.

AI-assisted autonomous learning may support autonomy and competence when students use AI tools to explore resources, clarify difficult content, and regulate their own learning. In this case, AI functions as a scaffold that expands students’ learning opportunities. However, the same tools may also weaken autonomy and competence when students depend on AI to define problems, generate answers, and complete academic tasks. In such situations, the learning process may appear autonomous but become externally guided in substance. Students may finish tasks, but they may feel less ownership over the learning outcome. This may increase their sense of reduced academic accomplishment.

Cognitive Load Theory offers a complementary explanation for this process. According to this theory, learning depends on the effective allocation of limited cognitive resources (Sweller, 2011). AI tools may reduce unnecessary cognitive load by organizing information and providing timely explanations. However, excessive reliance on AI may also reduce active processing, metacognitive monitoring, and effortful problem solving. Students may receive ready-made answers without fully engaging with the underlying reasoning. As a result, they may gain efficiency without developing deep understanding or mastery.

Hardiness can be interpreted within this self-determination framework. Hardiness includes commitment, control, and challenge (Kobasa, 1979). These characteristics help students remain engaged, perceive themselves as capable of influencing outcomes, and view difficulty as manageable. In this sense, hardiness supports autonomy and competence in learning. Students with higher hardiness are more likely to experience themselves as active agents when facing academic challenges. They are also more likely to maintain a sense of competence during difficult learning tasks.

AI-assisted autonomous learning may be related to hardiness because it changes students’ experience of autonomy, control, and challenge. When AI is used as a scaffold, students still need to make decisions, monitor progress, and solve problems. This may support the operation of hardiness. However, when AI replaces students’ own effortful engagement, students may have fewer opportunities to practice persistence, control, and challenge appraisal. As a result, hardiness may be less activated in learning contexts. Lower hardiness may then increase students’ vulnerability to reduced academic accomplishment.

Self-Determination Theory suggests a coherent pathway among the study variables. AI-assisted autonomous learning may reshape students’ autonomy and competence experiences. These experiences may be associated with hardiness as a psychological resource. Hardiness may further influence students’ academic self-evaluation, especially their sense of academic accomplishment. Therefore, hardiness is expected to mediate the relationship between AI-assisted autonomous learning and reduced academic accomplishment.

### 2.4 Hypothesis development

Based on Self-Determination Theory, AI-assisted autonomous learning may be negatively associated with hardiness when AI use weakens students’ authentic autonomy and competence experiences. Hardiness depends on students’ sense of commitment, control, and challenge. These characteristics are strengthened when students actively face difficulty and experience themselves as capable learners. However, when students rely heavily on AI to complete core learning tasks, they may have fewer opportunities to practice effortful control and challenge appraisal. Therefore, the following hypothesis are proposed:

*H1*: AI-assisted autonomous learning is negatively associated with hardiness.

*H2*: Hardiness is negatively associated with reduced academic accomplishment.

*H3*: AI-assisted autonomous learning is positively associated with reduced academic accomplishment.

*H4*: Hardiness mediates the relationship between AI-assisted autonomous learning and reduced academic accomplishment.

**Figure 1:**
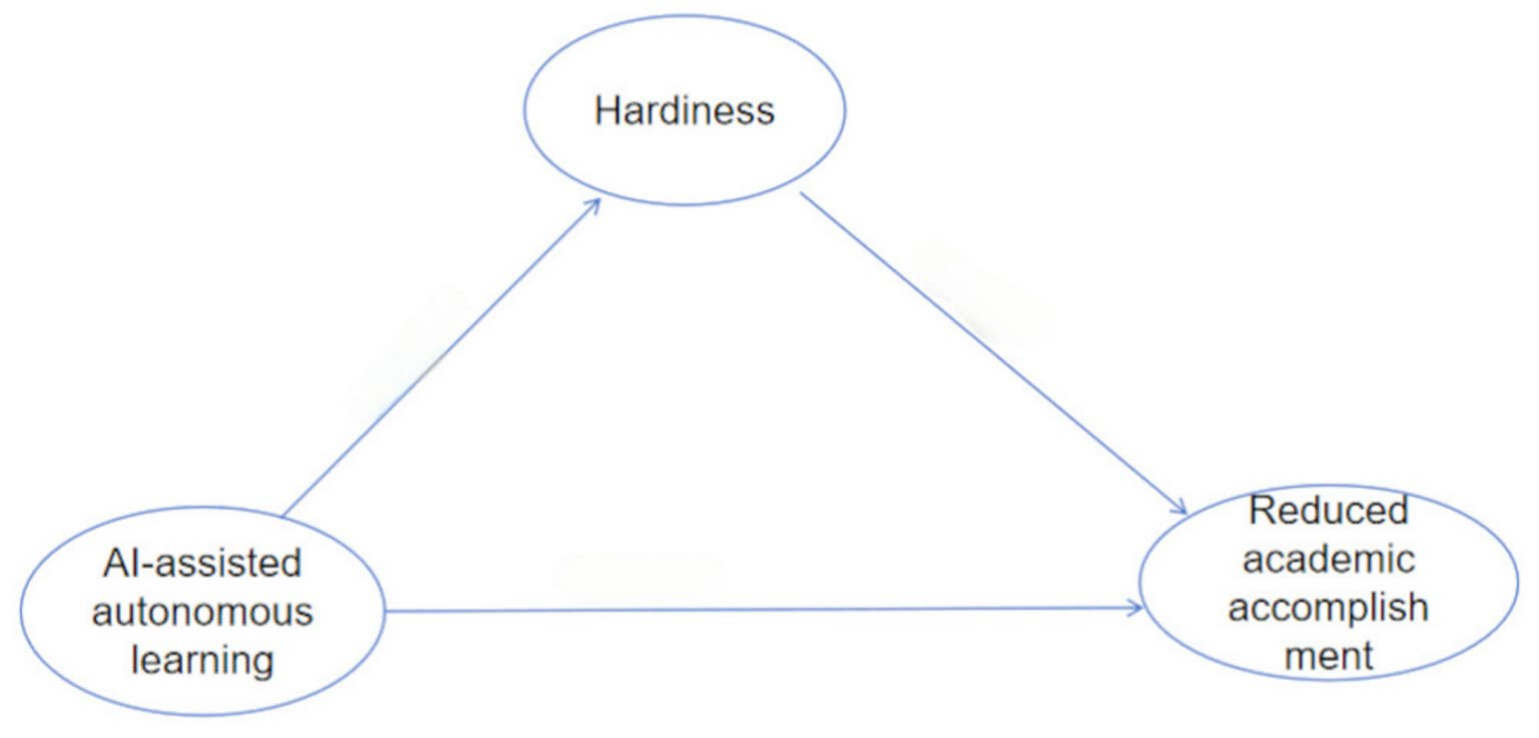
Conceptual model of the study. The model proposes that AI-assisted autonomous learning is directly associated with reduced academic accomplishment and indirectly associated with reduced academic accomplishment through hardiness.

The hypothesized relationships among AI-assisted autonomous learning, hardiness, and reduced academic accomplishment are presented in Figure 1.

## 3 Method

### 3.1 Participants

This study employed a questionnaire survey design and recruited students from a vocational college in China. The target population was vocational college students who had experience with AI-assisted learning in academic activities. To improve sample coverage within the participating institution, stratified cluster sampling was used.

Specifically, two to three majors were selected from three disciplinary categories: equipment manufacturing, electronics and information technology, and medicine and health. Within each selected major, one or two intact classes were sampled from each grade level (Years 1–3). This sampling procedure allowed the study to include students from different disciplinary backgrounds and grade levels. Data were collected in supervised classroom settings through an online questionnaire.

A total of 1,316 questionnaires were returned. After excluding invalid responses with missing data, logical inconsistencies, or substantial incompleteness, 1,264 valid questionnaires were retained, yielding an effective response rate of 96.0%. Of the valid participants, 546 were male (43.0%) and 698 were female (57.0%). In terms of grade level, 633 were first-year students, 379 were second-year students, and 252 were third-year students. By disciplinary background, 608 students were from science and engineering fields and 656 were from humanities and social science fields. Participation was voluntary, and written informed consent was obtained from all participants prior to data collection.

### 3.2 Procedure

The questionnaire was distributed via an anonymous online link, and the average completion time was approximately 8 to 12 min. All participants completed the survey independently during regular class hours under the supervision of trained research assistants.

To reduce potential common method bias, several procedural remedies were embedded in the data collection process. The questionnaire was completed anonymously, and participants were informed that there were no correct or incorrect answers. They were also told that their responses would be used only for academic research and would remain strictly confidential. During data collection, participants completed the survey independently, which helped minimize discussion or mutual influence. Reverse-scored items were included to reduce response pattern bias, and all measures were presented in a standardized order: demographic information, AI-assisted autonomous learning, hardiness, and academic burnout.

### 3.3 Instruments

#### 3.3.1 AI-assisted autonomous learning scale

AI-assisted autonomous learning was measured using the scale developed by Wang and Li (2024). The scale includes 27 items across seven dimensions: expectation confirmation, perceived usefulness, self-efficacy, continuance intention, satisfaction, usage willingness, and positive emotions. All items were rated on a 5-point Likert scale ranging from 1 (strongly disagree) to 5 (strongly agree). Higher total scores indicate a higher level of AI-assisted autonomous learning in academic activities.

#### 3.3.2 Hardiness scale

Hardiness was measured using the Chinese version of the Hardiness Scale revised by Lu and Liang (2008) based on Kobasa’s (1979) hardiness framework. The scale includes four dimensions: commitment, control, challenge, and resilience. All items were rated on a 5-point Likert scale, with response options ranging from 1 (strongly disagree) to 5 (strongly agree). Higher scores indicate higher levels of hardiness. Reverse-scored items were recoded before the analyses.

#### 3.3.3 Academic burnout scale

Academic burnout was measured using the College Student Academic Burnout Scale developed by Lian et al. (2005). The scale includes three dimensions: emotional exhaustion, behavioral disengagement, and reduced academic accomplishment. All items were rated on a 5-point Likert scale ranging from 1 (strongly disagree) to 5 (strongly agree). Higher scores indicate higher levels of academic burnout. Reverse-scored items were reverse-coded before the formal analyses.

The Academic Burnout Scale was used to assess the multidimensional structure of academic burnout. However, the structural model focused specifically on reduced academic accomplishment as the dependent variable. It is conceptually consistent with hardiness as a psychological resource related to persistence, control, and adaptive coping. By contrast, emotional exhaustion mainly reflects affective depletion and showed a weaker direct association with AI-assisted autonomous learning in the preliminary analyses.

### 3.4 Analysis plan

All statistical analyses were conducted using SPSS 26.0 and AMOS 26.0. Descriptive statistics and Pearson correlation analyses were first performed to examine the associations among the focal variables. Confirmatory factor analyses were then conducted to evaluate the measurement properties of the main study constructs.

Common method bias was further assessed using Harman’s single-factor test (Podsakoff et al., 2003). All measurement items were entered into an unrotated exploratory factor analysis. Following the commonly used criterion, common method bias was considered a concern if a single factor emerged or if the first unrotated factor explained more than 40% of the total variance.

Structural equation modeling (SEM) was used to test the hypothesized relationships among AI-assisted autonomous learning, hardiness, and reduced academic accomplishment. Maximum likelihood estimation was adopted, and model fit was evaluated using commonly reported indices, including the chi-square to degrees of freedom ratio (*χ*^2^/df), the Comparative Fit Index (CFI), the Tucker–Lewis Index (TLI), and the Root Mean Square Error of Approximation (RMSEA).

In the structural analyses, reduced academic accomplishment was treated as the primary outcome variable. This decision was based on both conceptual and empirical considerations. Conceptually, reduced academic accomplishment more directly reflects students’ evaluations of their learning competence and academic achievement, making it especially relevant to the psychological resource perspective adopted in this study. Empirically, this dimension showed acceptable reliability and stronger associations with AI-assisted autonomous learning and hardiness than the other burnout dimensions in the preliminary analyses. By contrast, emotional exhaustion primarily reflects affective depletion and showed a weaker direct association with AI-assisted autonomous learning.

The mediating effect of hardiness was tested using the bias-corrected bootstrap method with 5,000 resamples. Indirect effects were considered statistically significant when the 95% confidence interval did not include zero. Direct, indirect, and total effects were examined to clarify the mechanism linking AI-assisted autonomous learning to reduced academic accomplishment.

## 4 Results

### 4.1 Descriptive statistics and correlations

Before conducting the main analyses, common method bias was examined using Harman’s single-factor test. All measurement items were entered into an unrotated exploratory factor analysis. The results showed that the first unrotated factor explained 32.62% of the total variance, which was below the 40% threshold (Podsakoff et al., 2003). Therefore, common method bias was unlikely to seriously affect the findings of this study.

Descriptive statistics and Pearson correlation analyses were conducted to examine the preliminary associations among the study variables. The results are presented in Table 1. The mean scores of the seven AI-assisted autonomous learning dimensions ranged from 3.82 to 3.87, indicating relatively high levels of AI-assisted autonomous learning. The mean scores of the hardiness dimensions ranged from 2.36 to 2.40, suggesting moderate levels of hardiness. Among the academic burnout dimensions, reduced academic accomplishment had the highest mean score (*M* = 3.41, SD = 0.61).

**Table 1:** Correlation of the main variables.

Variables	1	2	3	4	5	6	7	8	9	10	11	12	13	14	15	16
1. Expectation confirmation	1	0.922***	0.913***	0.862***	0.855***	0.856***	0.816***	−0.116**	−0.159***	−0.14***	−0.124**	−0.141***	0.041	0.116**	0.175***	0.127***
2. Perceived usefulness		1	0.935***	0.886***	0.894***	0.898***	0.873***	−0.151***	−0.185***	−0.167***	−0.157***	−0.173***	0.042	0.15***	0.214***	0.152***
3. Satisfaction			1	0.889***	0.901***	0.898***	0.86***	−0.145***	−0.188***	−0.165***	−0.153***	−0.17***	0.037	0.16***	0.222***	0.156***
4. Continuance intention				1	0.893***	0.887***	0.842***	−0.147***	−0.161***	−0.133***	−0.12**	−0.148***	−0.005	0.099*	0.164***	0.09*
5. Willingness for autonomous learning					1	0.93***	0.909***	−0.163***	−0.2***	−0.171***	−0.165***	−0.183***	0.047	0.167***	0.223***	0.164***
6. Self-efficacy						1	0.927***	−0.164***	−0.204***	−0.182***	−0.169***	−0.188***	0.057	0.157***	0.222***	0.167***
7. Positive emotions							1	−0.19***	−0.209***	−0.194***	−0.191***	−0.205***	0.064	0.172***	0.227***	0.178***
8. Control								1	0.867***	0.864***	0.886***	0.954***	−0.097*	−0.265***	−0.569***	−0.353***
9. Challenge									1	0.891***	0.886***	0.954***	−0.107**	−0.296***	−0.63***	−0.391***
10. Commitment										1	0.888***	0.951***	−0.129***	−0.309***	−0.606***	−0.399***
11. Resilience											1	0.956***	−0.089*	−0.275***	−0.611***	−0.366***
12. Hardiness												1	−0.11*	−0.299***	−0.631***	−0.395***
13. Emotional exhaustion													1	0.73***	0.091*	0.86***
14. Behavioral disengagement														1	0.377***	0.897***
15. Reduced personal accomplishment															1	0.544***
16. Learning burnout																1
*M*	3.8671	3.8573	3.8291	3.8676	3.8275	3.8536	3.8165	2.3572	2.3784	2.3787	2.4043	2.3779	3.0388	3.1994	3.4114	3.1987
SD	0.76713	0.76131	0.76644	0.76383	0.7747	0.74632	0.78554	0.61835	0.60138	0.64304	0.61531	0.59016	0.73018	0.55937	0.60945	0.50128
Interaction	High	High	High	High	High	High	High	Moderate	Moderate	Moderate	Moderate	Moderate	Moderate	Moderate	Moderate	Moderate

*p < 0.05, **p < 0.01, ***p < 0.001.

The seven dimensions of AI-assisted autonomous learning were strongly and positively correlated with one another, with correlations ranging from 0.816 to 0.935, all *p*s < 0.001. They were also significantly and negatively correlated with hardiness, with correlations ranging from −0.141 to −0.205, all *p*s < 0.01. This pattern suggests that higher AI-assisted autonomous learning was associated with lower hardiness.

AI-assisted autonomous learning was not significantly correlated with emotional exhaustion. However, its seven dimensions were significantly and positively correlated with behavioral disengagement, reduced academic accomplishment, and total academic burnout. The correlations with reduced academic accomplishment ranged from 0.164 to 0.227, all *p*s < 0.001. These associations were stronger and more consistent than those with the other burnout dimensions, supporting the focus on reduced academic accomplishment as the primary outcome variable.

Hardiness was significantly and negatively correlated with academic burnout and its three dimensions. The strongest association was observed between hardiness and reduced academic accomplishment, *r* = −0.631, *p* < 0.001. This finding indicates that students with higher hardiness tended to report lower negative academic self-evaluation. Overall, the correlation pattern was consistent with the proposed hypotheses and provided preliminary support for the subsequent structural equation modeling analyses.

Therefore, the correlation pattern was consistent with the proposed hypotheses. AI-assisted autonomous learning was negatively associated with hardiness and positively associated with reduced academic accomplishment, whereas hardiness was negatively associated with reduced academic accomplishment. These preliminary findings provided a basis for the subsequent structural equation modeling analyses.

### 4.2 Measurement validity and reliability

Following the correlation analyses, the validity and reliability of the study measures were further examined. Confirmatory factor analyses and reliability analyses were conducted to evaluate the measurement quality of the main study constructs. The results are presented in Table 2. The measurement analyses focused on AI-assisted autonomous learning, hardiness, and reduced academic accomplishment, as these constructs were included in the structural model.

**Table 2:** Confirmatory factor analysis and reliability results for the study measures.

Measure	Factor structure	*χ^2^*	df	*χ^2^*/df	CFI	TLI	RMSEA	Cronbach’s *α*	Standardized loadings
AI-assisted autonomous learning	Second-order, 7 first-order factors	1728.95	317	5.454	0.949	0.944	0.084	0.991	0.95–0.99
Hardiness	Four-factor model	9.03	2	4.517	0.998	0.993	0.075	0.967	0.93–0.95
Reduced academic accomplishment	One fator model	39.92	9	4.436	0.973	0.955	0.074	0.824	0.60–0.75

AI-assisted autonomous learning was specified as a second-order factor with seven first-order factors. The standardized loadings reported for AI-assisted autonomous learning refer to the second-order factor loadings from the higher-order construct to the seven first-order factors. Hardiness was specified as a four-factor model. Reduced academic accomplishment was specified as a one-factor model using six items as indicators. The overall academic burnout construct was not modeled as a latent variable in the structural model.

For AI-assisted autonomous learning, a second-order factor model was specified according to the original seven-dimensional structure of the scale. The model showed an acceptable fit to the data, *χ*^2^ = 1728.95, df = 317, *χ*^2^/df = 5.454, CFI = 0.949, TLI = 0.944, and RMSEA = 0.084. The standardized factor loadings ranged from 0.95 to 0.99, and the total scale showed excellent internal consistency, with Cronbach’s *α* = 0.991.

For hardiness, a four-factor model was tested. The model showed a good fit to the data, *χ*^2^ = 9.03, df = 2, *χ*^2^/df = 4.517, CFI = 0.998, TLI = 0.993, and RMSEA = 0.075. The standardized factor loadings ranged from 0.93 to 0.95, and the total scale showed high internal consistency, with Cronbach’s *α* = 0.967.

For reduced academic accomplishment, a one-factor model was specified using its six items as indicators. The model showed an acceptable fit to the data, *χ*^2^ = 39.92, df = 9, *χ*^2^/df = 4.436, CFI = 0.973, TLI = 0.955, and RMSEA = 0.074. The standardized factor loadings ranged from 0.60 to 0.75, and the internal consistency was acceptable, with Cronbach’s *α* = 0.824.

These results indicated that the measurement models had acceptable reliability and validity. Therefore, the three constructs were retained for the subsequent structural equation modeling analysis. AI-assisted autonomous learning was modeled as a second-order latent variable, hardiness as a four-factor latent construct, and reduced academic accomplishment as a latent outcome variable. The overall academic burnout construct was not modeled in the SEM because the study focused specifically on reduced academic accomplishment.

### 4.3 Structural model testing

Based on the validated measurement model, a structural model linking AI-assisted autonomous learning, hardiness, and reduced academic accomplishment was estimated. The model showed a good fit to the data, with *χ*^2^ = 2286.124, *χ*^2^/df = 3.693, CFI = 0.949, TLI = 0.945, and RMSEA = 0.065. These results indicated that the proposed model was suitable for examining the direct and indirect relationships among the study variables. The standardized path coefficients are presented in Table 3.

**Table 3:** Path coefficient estimation results for structural models.

Paths	*β*	SE	CR	*p*
AI → RAA	0.112	0.030	3.242	0.001
AI → H	−0.189	0.033	−4.576	<0.001
H → RAA	−0.689	0.047	−15.541	<0.001

AI, AI-assisted autonomous learning; H, hardiness; RAA, reduced academic accomplishment.

Before interpreting the mediating pathway, the direct relationship between AI-assisted autonomous learning and reduced academic accomplishment was examined. The path from AI-assisted autonomous learning to reduced academic accomplishment was significant and positive, *β* = 0.112, SE = 0.030, CR = 3.242, *p* = 0.001. This result indicated that higher levels of AI-assisted autonomous learning were associated with higher levels of reduced academic accomplishment. Thus, H3 was supported.

The remaining paths in the structural model were also significant. AI-assisted autonomous learning was significantly and negatively associated with hardiness (*β* = −0.185, SE = 0.033, CR = −4.576, *p* < 0.001), supporting H1. Hardiness was significantly and negatively associated with reduced academic accomplishment (*β* = −0.689, SE = 0.047, CR = −15.541, *p* < 0.001), supporting H2. These findings indicated that students with higher AI-assisted autonomous learning tended to report lower hardiness, and students with higher hardiness tended to report lower reduced academic accomplishment.

**Figure 2:**
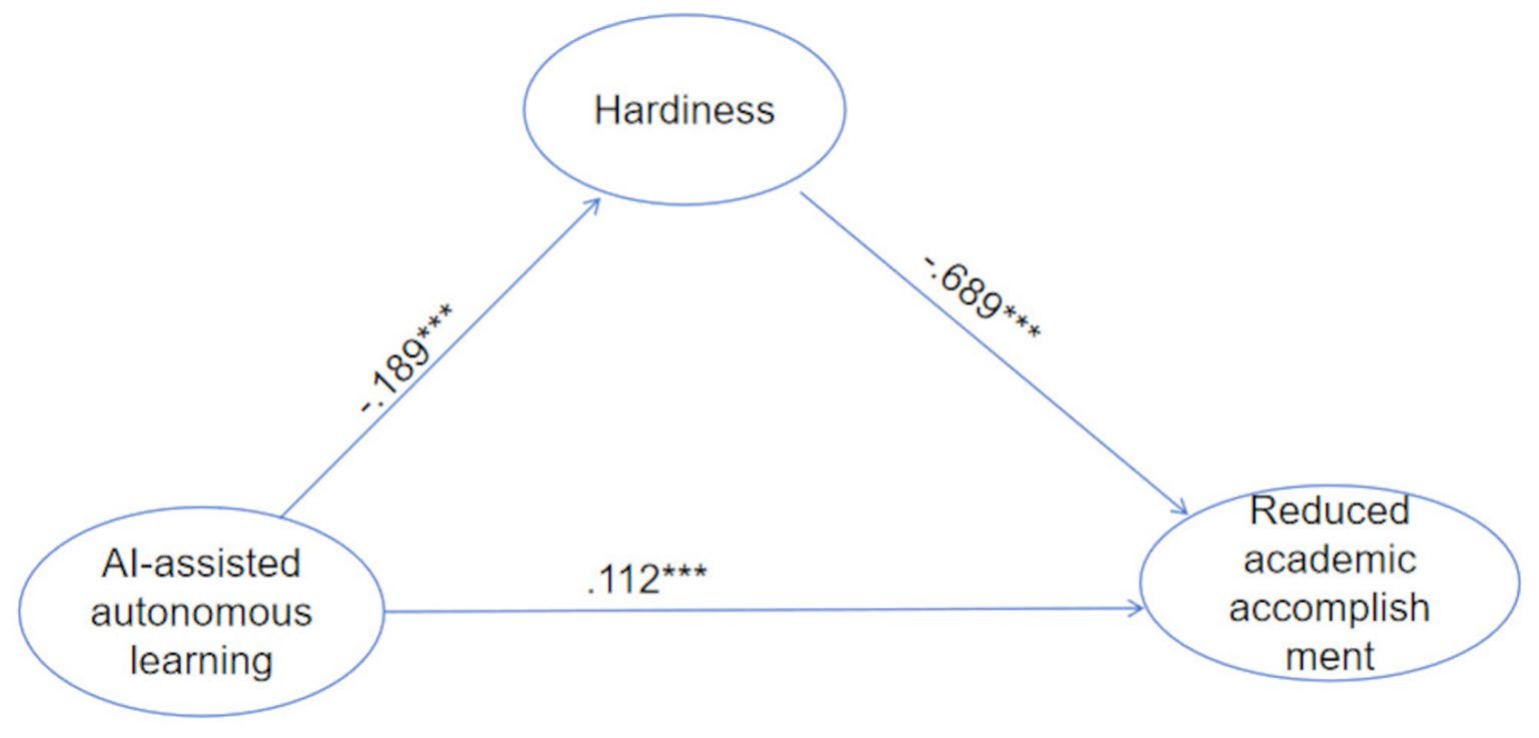
Structural model with standardized estimates. Standardized coefficients are reported. ***p < 0.001.

Therefore, the structural model supported the proposed direct relationships among AI-assisted autonomous learning, hardiness, and reduced academic accomplishment. The significant paths from AI-assisted autonomous learning to hardiness and from hardiness to reduced academic accomplishment also provided the basis for testing the mediating effect of hardiness. The final structural model with standardized estimates is presented in Figure 2.

### 4.4 Mediating effect test

The bias-corrected Bootstrap method (5,000 resamples) was used to test the mediating effect. The results are presented in Table 4.

**Table 4:** Bootstrap test results for mediation effects.

Path	Effect type	*B*	95% Bootstrap CI	Significant
AI → RAA	Total effect	0.209	[0.100, 0.322]	Significant
AI → RAA	Direct effect	0.098	[0.024, 0.182]	Significant
AI → H → RAA	Indirect effect	0.111	[0.043, 0.184]	Significant

B represents the unstandardized effect estimate. Confidence intervals were estimated using the bias-corrected bootstrap method with 5,000 resamples. The indirect effect was considered significant when the 95% confidence interval did not include zero. AI, AI-assisted autonomous learning; H, hardiness; RAA, reduced academic accomplishment.

The total effect of AI-assisted autonomous learning on reduced academic accomplishment was significant (*B* = 0.209, 95% CI [0.100, 0.322]). After hardiness was included in the model, the direct effect of AI-assisted autonomous learning on reduced academic accomplishment remained significant (*B* = 0.098, 95% CI [0.024, 0.182]). The indirect effect through hardiness was also significant (*B* = 0.111, 95% CI [0.043, 0.184]). None of the confidence intervals included zero, indicating that both the direct and indirect effects were statistically significant.

The indirect effect accounted for approximately 53.1% of the total effect. This result suggests that the association between AI-assisted autonomous learning and reduced academic accomplishment was transmitted to a substantial extent through hardiness. Because both the direct effect and the indirect effect were significant, hardiness was identified as a partial mediator in this relationship. H4 is supported. The relatively larger indirect effect further underscores the importance of hardiness as a psychological mechanism linking AI-assisted autonomous learning to reduced academic accomplishment.

## 5 Discussion

### 5.1 Key research findings

From a psychological resource perspective, this study examined the relationships among AI-assisted autonomous learning, hardiness, and reduced academic accomplishment among vocational college students. The results showed that AI-assisted autonomous learning was negatively associated with hardiness and positively associated with reduced academic accomplishment. In addition, hardiness partially mediated the relationship between AI-assisted autonomous learning and reduced academic accomplishment. Taken together, these findings suggest that the implications of AI-assisted autonomous learning may extend beyond learning efficiency and convenience to students’ psychological resources and academic self-evaluation. This overall pattern is consistent with the central argument advanced in the introduction and literature review, namely that AI-assisted autonomous learning should be understood not only as a technological practice, but also as a learning context with potential psychological consequences.

One notable finding is that AI-assisted autonomous learning was negatively associated with hardiness. Existing research has often emphasized the educational advantages of artificial intelligence, including improved learning efficiency, personalized support, and more positive learning experiences (Jagtap, 2025; Liu et al., 2025; Wang et al., 2024; Xu et al., 2024). The present findings do not deny these potential benefits. However, they also suggest that when AI-assisted autonomous learning becomes highly embedded in students’ self-directed study, it may be associated with lower levels of commitment, control, and challenge orientation. This result is broadly in line with previous concerns that excessive reliance on generative AI may weaken independent thinking, reduce active knowledge construction, and encourage more superficial learning strategies (Hendrycks et al., 2023; Rong and Yu, 2023; Zhai et al., 2024; Dong et al., 2025). In this sense, the present study extends prior literature by showing that the effects of AI-assisted autonomous learning may involve not only behavioral or performance-related outcomes, but also changes in internal psychological resources.

This pattern also resonates with the earlier discussion of hardiness as a psychological resource. Previous studies have shown that hardiness helps individuals maintain engagement under stress and serves as a protective factor against burnout and maladaptive academic outcomes (Nuradilla, 2024; Amelia et al., 2025; Fauziah and Affandi, 2025). The present results extend these findings into an AI-mediated learning context. When students increasingly rely on AI to generate answers, organize responses, and reduce task difficulty, they may have fewer opportunities to tolerate frustration, sustain effort, and solve problems independently. Over time, this may weaken the conditions that support the development or mobilization of hardiness. Thus, the present study not only confirms the importance of hardiness in academic adaptation, but also identifies AI-assisted autonomous learning as a contextual factor that may be related to its reduction.

Relatedly, AI-assisted autonomous learning was positively associated with reduced academic accomplishment. This finding indicates that students who reported higher levels of AI-assisted autonomous learning tended to report stronger negative evaluations of their academic competence and learning effectiveness. Reduced academic accomplishment is especially relevant in AI-supported learning contexts because students may complete academic tasks more efficiently without necessarily experiencing genuine mastery. In other words, task completion supported by AI may not always translate into a strong sense of personal academic competence. This finding extends prior AI-related research by shifting attention from technology acceptance, performance, and short-term motivation to students’ academic self-evaluation.

As noted in the literature review, reduced academic accomplishment reflects students’ negative evaluation of their learning competence and academic performance (Lian et al., 2005; Maslach and Jackson, 1981). It is especially relevant in technology-mediated environments where task completion may become easier but authentic experiences of mastery may become weaker. The present finding is compatible with earlier concerns that heavy reliance on AI may reduce deep processing, meaningful engagement, and opportunities for independent problem solving (Dong et al., 2025; Lin and Chen, 2024). In other words, although AI-assisted autonomous learning may help students complete academic tasks more efficiently, it may also make some students less certain that their academic success reflects their own understanding and competence. This may, in turn, contribute to stronger feelings of reduced academic accomplishment.

This result is particularly meaningful because prior AI-related research has more often focused on acceptance, performance, motivation, or short-term emotional responses (Rong and Yu, 2023; Zhang et al., 2024), whereas less attention has been paid to academic self-evaluation as an outcome. The present study therefore extends the discussion by showing that AI-assisted autonomous learning may be linked to a more self-evaluative dimension of academic adaptation. This is also consistent with the rationale presented in Section 1.1.2, where reduced academic accomplishment was treated not merely as one burnout dimension among others, but as an indicator of weakened academic functioning and fragile self-perceived competence in learning. In this respect, the present findings provide empirical support for focusing specifically on reduced academic accomplishment in AI-mediated learning contexts.

Taken a step further, the mediation analysis showed that hardiness partially mediated the relationship between AI-assisted autonomous learning and reduced academic accomplishment. This result supports the indirect pathway proposed in Section 1.1.4 and is consistent with the psychological resource perspective guiding the study. Conservation of Resources Theory suggests that students rely on internal resources to maintain adaptation under academic demands (Hobfoll and Lilly, 1993), whereas Self-Determination Theory emphasizes the importance of autonomy and competence for psychological functioning (Ryan and Deci, 2000). Cognitive Load Theory further implies that when external systems handle increasingly complex tasks, students may invest less effort in reflection, metacognitive monitoring, and active problem solving (Sweller, 2011). The present mediation result is broadly consistent with these theoretical expectations. It suggests that AI-assisted autonomous learning may be associated with reduced academic accomplishment partly because it is related to lower hardiness, that is, lower commitment, control, and challenge orientation in learning.

At the same time, the mediation was only partial rather than complete. This result is theoretically important because it suggests that hardiness is an important, but not the only, mechanism linking AI-assisted autonomous learning to reduced academic accomplishment. This may help explain why the direct association between AI-assisted autonomous learning and reduced academic accomplishment remained significant after hardiness was included in the model. Therefore, the present findings support the role of hardiness as a meaningful psychological pathway, while also indicating that academic adaptation in AI-assisted learning environments is likely shaped by multiple interacting mechanisms.

Overall, the findings of this study both align with and extend the existing literature. They align with prior concerns that overreliance on AI may weaken deep engagement, self-regulated learning, and psychologically meaningful academic development (Hendrycks et al., 2023; Rong and Yu, 2023; Zhai et al., 2024; Dong et al., 2025). At the same time, they extend prior research by empirically linking AI-assisted autonomous learning to hardiness and reduced academic accomplishment within a single explanatory model. In this sense, the present study provides more specific evidence that the educational consequences of AI may operate not only through external learning support, but also through the restructuring of internal psychological resources that support academic adaptation.

### 5.2 Psychological mechanisms in the AI learning context

The present findings can be more fully understood from self-determination theory. As discussed earlier, academic adaptation depends not only on the use of external tools, but also on students’ experiences of autonomy, competence, and agency (Ryan and Deci, 2000). From this perspective, AI-assisted autonomous learning should not be viewed merely as a technical aid that improves efficiency. Rather, it can also be understood as a learning context that may reshape how students experience challenge, effort, control, and academic self-evaluation. This interpretation is consistent with the argument developed in the literature review that the educational implications of AI may extend beyond observable learning behaviors to the psychological conditions that support longer-term adaptation.

Self-Determination Theory provides a useful starting point for interpreting this pathway (Ryan and Deci, 2000). AI-assisted autonomous learning may support autonomy and competence when it functions as a scaffold that helps students clarify difficult content, organize learning materials, and regulate their own learning process. However, when learning is increasingly guided by external technological systems, students may have less room for independent effort, self-directed mastery, and personally meaningful problem solving. Under such conditions, academic tasks may still be completed successfully, but the sense that “I achieved this through my own understanding and effort” may become weaker. This may help explain why AI-assisted autonomous learning was positively associated with reduced academic accomplishment in the present study. In other words, the issue may not be task completion itself, but whether students continue to perceive themselves as competent and agentic learners while using AI.

This interpretation also helps explain the negative association between AI-assisted autonomous learning and hardiness. Hardiness reflects commitment, control, and challenge orientation. It can be understood as a personal psychological resource that supports resistance to stress and promotes further resource gain (Bartone and Roche, 2023; Chykhantsova, 2023). In academic settings, the development of hardiness depends on repeated experiences of challenge, uncertainty, and active problem solving. Learners need opportunities to persist through difficulty and gradually build a sense of control over demanding tasks. However, generative AI often provides instant answers, optimized learning paths, and highly structured support (Gkintoni et al., 2025). Although these functions may improve learning efficiency, they may also reduce learners’ opportunities to practice self-regulation, persistence, and challenge appraisal. Over time, this shift in learning experience may weaken the conditions needed for the development or activation of hardiness. In this sense, the negative association between AI-assisted autonomous learning and hardiness found in the present study is broadly compatible with a resource-based interpretation.

Cognitive Load Theory provides further support for the relationship between AI-assisted autonomous learning and reduced academic accomplishment (Sweller, 2011). When learners rely heavily on external systems for problem solving, deep cognitive processing, reflection, and metacognitive monitoring may decline. In AI-assisted learning contexts, students may increasingly prioritize rapid answer acquisition over iterative knowledge construction. Although this can make learning feel smoother and more efficient in the short term, it may also weaken authentic experiences of mastery. As argued in the literature review, reduced academic accomplishment is closely tied to students’ negative evaluation of their own academic competence and performance. If students receive answers without engaging sufficiently in effortful thinking, they may complete tasks without developing a strong sense of personal mastery. This may, over time, make them more vulnerable to reduced academic accomplishment. The present findings are therefore broadly consistent with previous concerns that generative AI may, under certain conditions, be linked to burnout-related outcomes and weaker academic self-evaluation (Dong et al., 2025; Lin and Chen, 2024).

Viewed together, these theoretical perspectives help explain why hardiness functioned as a partial mediator rather than a complete one. The present findings suggest that AI-assisted autonomous learning may be associated with reduced academic accomplishment partly because it is related to lower hardiness, that is, lower commitment, control, and challenge orientation in learning. At the same time, the mediation was only partial, indicating that hardiness is an important, but not the only, mechanism involved. As already suggested in the original discussion, other factors may also contribute, such as learning strategies, metacognitive engagement, cognitive load, or perceived academic control. This may help explain why the direct association between AI-assisted autonomous learning and reduced academic accomplishment remained significant after hardiness was included in the model. Thus, the present study supports the value of a psychological resource perspective, while also indicating that academic adaptation in AI-assisted learning environments is likely shaped by multiple interrelated processes.

### 5.3 Theoretical contributions

This study makes several theoretical contributions to the literature on artificial intelligence in education. To begin with, it extends current discussions of AI-assisted learning beyond the dominant emphasis on efficiency, personalization, and academic support. Existing studies have largely highlighted the benefits of artificial intelligence for improving learning performance, enhancing learning motivation, and supporting positive learning experiences (Jagtap, 2025; Liu et al., 2025; Wang et al., 2024; Xu et al., 2024). At the same time, other scholars have raised concerns about excessive reliance on AI, including weakened independent thinking, reduced deep engagement, and greater dependence on externally generated solutions (Hendrycks et al., 2023; Rong and Yu, 2023; Zhai et al., 2024). Building on these two strands of literature, the present study contributes by showing that AI-assisted autonomous learning should be understood not only as a technological practice with instrumental benefits, but also as a learning context that may be associated with students’ psychological resources and academic adaptation. In this respect, the study broadens the theoretical scope of AI-in-education research from performance-oriented outcomes to psychologically meaningful developmental outcomes.

Building on this broader perspective, a second contribution of the study is that it identifies hardiness as a meaningful psychological mechanism in AI-assisted learning contexts. Previous studies have consistently treated hardiness as an important protective resource that helps individuals remain committed, maintain control, and interpret difficulty as a challenge rather than a threat (Kobasa, 1979; Nuradilla, 2024; Amelia et al., 2025; Fauziah and Affandi, 2025). The present study extends this line of research by examining hardiness in an AI-mediated learning context. From the perspective of Self-Determination Theory, hardiness can be understood as a psychological resource closely related to students’ experiences of agency, control, and competence in learning (Ryan and Deci, 2000). The finding that hardiness partially mediated the relationship between AI-assisted autonomous learning and reduced academic accomplishment provides empirical support for this argument. Therefore, the present study does more than document a direct association between AI-assisted autonomous learning and academic adaptation. It also demonstrates that this relationship may operate through students’ internal psychological resource system. In this way, the study strengthens the value of a psychological resource perspective for understanding academic adaptation in AI-mediated learning environments.

Extending this line of reasoning, a further contribution of the study lies in its specific focus on reduced academic accomplishment as an outcome. Prior research on AI in education has more often examined technology acceptance, academic performance, learning engagement, or short-term emotional experiences (Rong and Yu, 2023; Zhang et al., 2024). By contrast, less attention has been paid to students’ self-evaluative academic functioning. As discussed earlier, reduced academic accomplishment reflects students’ negative evaluation of their own academic competence and performance and represents a core dimension of academic burnout (Maslach and Jackson, 1981; Lian et al., 2005). This focus is theoretically important because AI-assisted learning environments may enable students to complete tasks more efficiently while simultaneously weakening authentic experiences of mastery, deep processing, and self-generated competence (Dong et al., 2025; Lin and Chen, 2024). By centering reduced academic accomplishment rather than treating academic burnout as a broad undifferentiated construct, the present study refines how maladaptive academic outcomes are conceptualized in AI-related educational research. Taken together, these contributions suggest that AI-assisted autonomous learning should be examined not only in terms of what it helps students do, but also in terms of how it is associated with the psychological resources through which students interpret and evaluate their own academic functioning.

### 5.4 Practical implications

The findings of this study have important practical implications for the responsible integration of artificial intelligence in higher education. As AI-assisted autonomous learning becomes increasingly common, its educational value should not be evaluated solely in terms of efficiency, convenience, or task completion. Rather, universities also need to consider how AI use may be related to students’ psychological resources and academic self-evaluation. This implication follows directly from the present finding that AI-assisted autonomous learning was associated not only with reduced academic accomplishment, but also with lower hardiness. In this sense, the responsible use of AI in higher education requires a broader educational perspective that balances technological support with students’ longer-term academic adaptation.

A first implication concerns instructional design. Existing studies have highlighted the benefits of AI for personalized support, learning efficiency, and improved learning experiences (Jagtap, 2025; Xu et al., 2024), and these functions should not be ignored. At the same time, the present findings suggest that AI should not be treated merely as a tool for rapid task completion. Teachers should intentionally retain moderately challenging tasks in AI-supported courses. They should also design learning activities that still require students to explain, reflect, revise, and make decisions independently. Such arrangements may help preserve students’ sense of control, persistence, and ownership while still allowing them to benefit from AI-supported learning.

Building on this instructional implication, a second implication concerns student support and developmental guidance. Because hardiness emerged as an important psychological mechanism in the present study, universities should pay greater attention to strengthening students’ psychological resilience in increasingly technology-mediated learning environments. This does not mean discouraging AI use altogether. Rather, it suggests that academic advising, counseling services, and student development programs should help students build challenge orientation, control beliefs, and sustained engagement while using AI tools. Such support is especially important in contexts where students may become highly dependent on immediate answers and externally structured solutions. By strengthening psychological resources alongside digital competence, universities may be better able to support adaptive patterns of AI-assisted learning.

Extending these implications further, AI tools should be integrated as supportive scaffolds rather than replacement systems. This point is also consistent with the theoretical discussion developed earlier from the perspectives of Self-Determination Theory and Cognitive Load Theory. In practice, AI should be used to stimulate students’ thinking rather than to replace it. For example, AI systems may be designed or used to provide prompting questions, step-by-step hints, alternative perspectives, or reflective feedback rather than complete solutions. Such an approach may help preserve deep cognitive processing, reduce excessive cognitive outsourcing, and support students’ continued experience of competence through their own effort. In this way, the educational value of AI lies not only in what it delivers, but also in how it shapes the learner’s role in the learning process.

At a broader level, the present findings also point to the need for clearer institutional guidance on responsible AI use. Because the direct association between AI-assisted autonomous learning and reduced academic accomplishment remained significant even after hardiness was included in the model, the educational influence of AI is likely to operate through multiple pathways. Universities should therefore move beyond simple encouragement or prohibition and develop more balanced governance strategies. These may include clearer usage guidelines, stronger digital literacy education, explicit discussion of both the benefits and risks of AI-supported learning, and institutional norms that encourage students to use AI as a learning aid rather than as a substitute for intellectual effort. Overall, the integration of AI in higher education requires a careful balance between efficiency gains and students’ psychological development.

### 5.5 Limitations and future research

Despite its theoretical and empirical contributions, this study has several limitations that should be acknowledged. Recognizing these limitations is important for interpreting the findings appropriately and for identifying directions for future research. The present results provide preliminary evidence for the psychological implications of AI-assisted autonomous learning in higher education, but they should be understood within the boundaries of the current research design.

A first limitation concerns the cross-sectional design. Although structural equation modeling was used to test the hypothesized relationships, causal inferences cannot be drawn with certainty. It remains possible that reverse or reciprocal relationships exist among the variables. Future research should therefore adopt longitudinal, cross-lagged, or experimental designs to clarify the direction and developmental dynamics of these relationships. Such work would help determine whether the present pathway is stable over time and whether AI-related learning behaviors actively contribute to changes in students’ psychological resources and academic self-evaluation.

A second limitation lies in the reliance on self-report data. All variables in the present study were measured through self-report questionnaires, which may increase the risk of common method bias. Although several procedural remedies were adopted, including anonymous participation and confidentiality instructions, the influence of single-source data cannot be fully ruled out. Future studies could combine self-report measures with behavioral indicators, learning analytics, platform usage records, teacher ratings, or peer evaluations. Such multi-method designs would improve the objectivity and robustness of the findings and would be especially useful in AI-assisted learning research, where actual usage patterns may not always correspond closely to students’ subjective perceptions.

A further limitation concerns the generalizability of the findings. Participants were recruited from a single vocational college in China, which may restrict the external validity of the results. The relationships among AI-assisted autonomous learning, hardiness, and reduced academic accomplishment may differ across institutional types, disciplinary fields, grade levels, or cultural settings. Future research should include more diverse samples from multiple institutions and broader educational settings to determine whether the present findings remain stable across different student populations.

The study is also limited by its specific outcome focus. Reduced academic accomplishment was selected because it showed stronger associations with AI-assisted autonomous learning and hardiness than the other burnout dimensions and because it was conceptually consistent with the psychological resource perspective adopted in this study. However, the mechanisms underlying emotional exhaustion and behavioral disengagement were not examined in depth. It is possible that different dimensions of academic burnout are linked to AI-assisted autonomous learning through different pathways. Future research could therefore construct comparative models to test whether distinct burnout dimensions are shaped by different psychological mechanisms. This would help refine current understanding of how AI-assisted learning relates to different forms of academic maladaptation.

Finally, the present study mainly adopted a psychological resource perspective. Although this perspective was useful for explaining the mediating role of hardiness, it is unlikely to provide a complete account of academic adaptation in AI-assisted learning contexts. As suggested in both the literature review and the present discussion, other cognitive and social factors may also be involved, including cognitive load, learning strategies, metacognitive engagement, perceived academic control, and social comparison processes. Future studies could integrate multiple theoretical perspectives to develop a more comprehensive framework for understanding academic adaptation in the AI era. In this way, subsequent research may move beyond a single-path explanation and provide a more nuanced account of how AI reshapes students’ academic functioning. Overall, the present study offers preliminary evidence rather than a final conclusion, and its findings require further replication and extension in larger, more diverse, and methodologically richer studies.

## 6 Conclusion

This study examined the relationship between AI-assisted autonomous learning and reduced academic accomplishment among vocational college students, with a focus on the mediating role of hardiness. The findings showed that AI-assisted autonomous learning was negatively associated with hardiness and positively associated with reduced academic accomplishment, while hardiness was negatively associated with reduced academic accomplishment and partially mediated this relationship. These results suggest that AI-assisted autonomous learning is related not only to learning efficiency and academic support, but also to students’ psychological resources and competence-related academic self-evaluation. The study extends AI-in-education research by highlighting hardiness as a psychological mechanism in AI-assisted learning contexts. As AI becomes increasingly embedded in higher education, educators should integrate AI as a scaffold rather than a substitute for active engagement, while supporting students’ resilience, agency, and sense of academic competence.

## Data Availability

The original contributions presented in the study are included in the article/supplementary material, further inquiries can be directed to the corresponding author.
