# Correction: AI-assisted autonomous learning and reduced academic accomplishment in vocational higher education: the mediating role of hardiness

**DOI:** 10.3389/fpsyg.2026.1947149

**Published:** 2026-08-06

**Authors:** Wenli Wang, Qiuyan Zhang

**Affiliations:** 1 Human Resources Department, Xiamen Institute of Technology, Xiamen, China; 2 Faculty of Education and Livelihood, Xiamen City University, Xiamen, China

**Keywords:** academic burnout, AI-assisted autonomous learning, hardiness, psychological resources, reduced academic accomplishment

An incorrect number was provided for the 2026 University-level Scientific Research Fund Project of Xiamen Institute of Technology (Artificial Intelligence Empowerment Special Project). The correct number is KYZXQTS202610.

The original version of this article has been updated.

